# Comparison of T2 and T3 sympathectomy for compensatory sweating on palmar hyperhidrosis

**DOI:** 10.1097/MD.0000000000006697

**Published:** 2017-04-21

**Authors:** Atila Turkyilmaz, Sami Karapolat, Kubra Nur Seyis, Celal Tekinbas

**Affiliations:** Department of Thoracic Surgery, Karadeniz Technical University Medical School, Trabzon, Turkey.

**Keywords:** hyperhidrosis, quality of life, sweating, sympathectomy

## Abstract

**Background:**

An otherwise successfully performed endoscopic thoracic sympathectomy (ETS) to treat palmar hyperhidrosis (PH) often has a serious side effect: compensatory sweating (CS). This side effect occurs in other parts of the body to a disturbing extent. The objective of this study is to determine whether there is a relationship between the level of ETS performed on patients with PH, and the occurrence and severity of postoperational CS.

**Methods:**

Between January 2014 and January 2015, ETS procedures were performed on 25 randomly selected consecutive subjects (group A) at T2 level, and on another 25 subjects (group B) at T3 level, who all felt severely handicapped due to PH. All subjects were assessed in terms of their demographic characteristics including gender and age, as well as postoperative complications, short-term results, side effects, recurrence of symptoms, and long-term results.

**Results:**

The symptoms disappeared in all subjects in short-term, and no recurrence was seen in their short or long-term follow-ups. At the end of year one, CS developed at a rate of 12% in group A and 8% in group B, particularly in their back and abdominal regions. The overall satisfaction with the procedure in year one was 96% in group A and 100% in group B.

**Conclusion:**

When an ETS performed at T2 or T3 level for PH involves only the interruption of the sympathetic chain, with a limitation on the range of dissection and avoidance of any damage to ganglia, sweating is stopped completely. No recurrence of PH is encountered, and CS develops only at low rates and severities.

## 1 Introduction

Palmar hyperhidrosis (PH) is a benign and idiopathic sympathetic disorder of excessive sweating. PH negatively affects the daily, social, emotional, and professional lives of patients particularly when sweating is excessive. This may lead to social withdrawal, social phobia, and even depression with a negative impact on quality of life. With a prevalence ranging from 0.3% to 4.5% in the general population, PH occurs equally in men and women and conservative therapy is usually unsatisfactory.^[1]^

Endoscopic thoracic sympathectomy (ETS) has been used increasingly as a thoracic surgery procedure in recent years. It has become a standard, well-tolerated treatment for PH as it is a safe, effective, and minimally invasive method.^[1,2]^ However, the symptoms may relapse in some patients after their ETS, and more importantly side effects such as compensatory sweating (CS) can occur in different regions of the body. CS is the most common and undesirable long-term complication of ETS, which occurs at a rate of between 3% and 98%, and is considered to be the “quality marker” of an ETS.^[3–5]^ It is generally believed that as the level at which the sympathetic chain is interrupted is lowered, the risk of postoperative recurrence increases, but the rate of CS decreases.^[2,3,6–9]^ However, there is no consensus in the literature regarding the relationship between the prevalence of CS, the ganglion level, the number of ganglion levels interrupted, and the different operative methods used. Results vary from study to study.^[1,3,10,11]^ For this reason, the present randomized study was performed to determine the relationship (if any) between the level of ETS and the occurrence and severity of CS.

## 2 Methods

### 2.1 Population and study design

Between January 2014 and January 2015, T2 level ETS procedures were performed at the Department of Thoracic Surgery in Karadeniz Technical University Medical School, on 25 randomly selected consecutive subjects (group A) and T3 level ETS on another 25 subjects (group B). Both groups felt severely handicapped due to PH and conservative treatments had failed. An informed consent form was signed by each of the patients before the operation, after careful explanation of the procedure and the goals of the study. All of the patients were unaware the level of ETS performed. All of the procedures were performed by the same surgical team, and standard surgical techniques were used throughout the study. All subjects were assessed in terms of their demographic characteristics including gender and age, as well as postoperative complications, short-term results, side effects, recurrence of symptoms, and long-term results. In addition, all subjects were examined and assessed at postoperative month 1 and at the end of year 1, and their ratings of satisfaction with the operation were recorded. The study began in 2014 upon the approval of the local ethics committee of Karadeniz Technical University Medical School.

### 2.2 Surgical procedure

**Figure 1:**
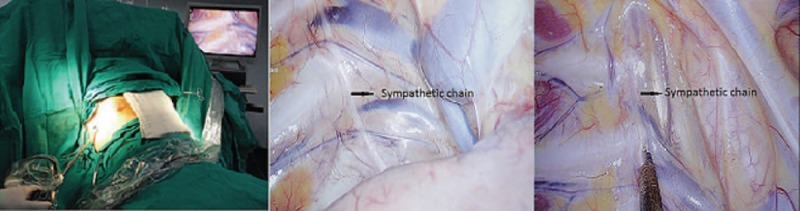
The videothoracoscope was inserted from the 3rd intercostal space while the subject was in a supine semisitting position with abduction of both arms. The sympathetic chain was identified and transected with electrocauterization.

**Figure 2:**
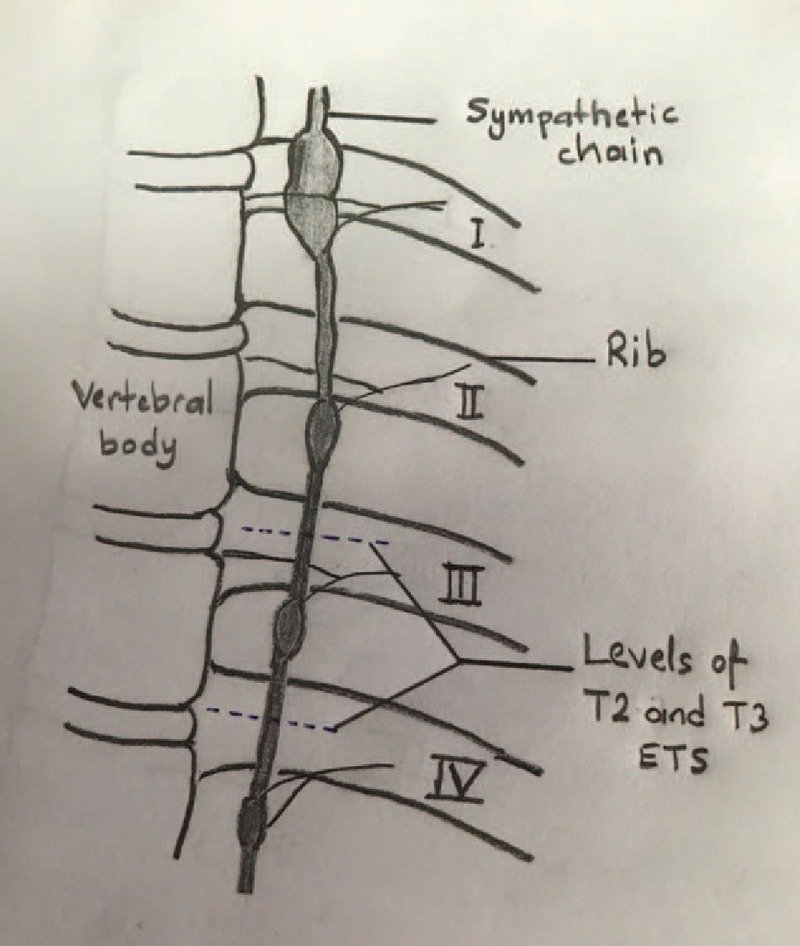
T2 and T3 levels from where the sympathetic chain and electrocauterization procedures were performed.

Under general anesthesia, all patients were administered a single-session bilateral ETS operation in the supine semisitting position with abduction of both arms, using a single port of a 1-cm incision in the 3rd intercostal space from the lateral of the pectoralis major muscle on the anterior axillary margin. After introduction of a 30° videothoracoscope, the parietal pleura was opened, the sympathetic chain and accessory nerve fibers were transected over the 2nd ribs (T2) in group A, and over the 3rd ribs (T3) in group B with electrocauterization (Figs. 1 and 2). Although the lungs were expanded by the anesthesiologist in all patients, the air in the pleural space was evacuated, and the incisions were closed without placing any thoracic drain. Postoperative chest radiography was performed routinely to exclude any pulmonary or pleural abnormalities.

### 2.3 Statistical analysis

All statistical data analyses were performed using the SPSS statistics software (SPSS Inc., Chicago, IL), version 15.0. The numerical variables were shown as mean ± SD. The chi-square test was used for comparisons. A value of *P* < .05 was considered statistically significant.

## 3 Results

Of the 25 subjects in group A, 18 were male (72.0%) and 7 were female (28.0%), with their mean age being 21.8 ± 3.8 years. Of the 25 subjects in group B, 15 were male (60.0%) and 10 were female (40.0%), with their mean age being 22.8 ± 4.9 years. There was no statistically significant difference between the groups in terms of age (*P* = .448).

There was no preoperative mortality or any conversion to an open thoracotomy in either of the groups. No complications developed in any of the patients in the early postoperative period. The median length of hospital stay was 1.3 ± 0.3 days in group A and 1.4 ± 0.3 days in group B.

There was dryness and increased heat, but no sweating in both hands of the subjects in the 2 groups immediately after the operation, showing an obvious improvement. All subjects were assessed, interviewed, and physically examined at the end of postoperative month 1, and the same positive effects were seen to continue with no recurrence of symptoms. Excessively dry hands occurred in 1 subject in group A (4%) and 2 subjects in group B (8%) during that period, but this condition was not seriously irritating. This side effect occurred without causing a statistically significant difference between group A and group B (*P* = .512). The patients were interviewed regarding their satisfaction at the end of month 1 to reveal their perceptions of the immediate effects of the operation. A total of 100% of group A and group B patients rated the operation outcomes as excellent.

The median follow-up time was 13 ± 2.1 months in group A and 13.6 ± 2.2 months in group B. All of the patients were invited to the hospital at the end of the 1st year to be interviewed and physically examined. The dryness and increased heat in hands continued in both groups, but there was neither sweating nor recurrence of symptoms. Although the dryness continued in 1 subject in group A and 2 subjects in group B, who were found to have excessive dryness at the end of month 1, there was a decrease in its severity. An assessment of the subjects for other side effects showed that there was CS in the back and abdominal regions of 3 subjects in group A (12%) and 2 subjects in group B (8%). There was no statistically significant difference between the groups with respect to CS (*P* = .637). Of these subjects, a 28-year-old male patient in group A had a severely irritating CS in his abdominal region, which caused patient dissatisfaction. He stated that he regretted having undergone the operation and he would not have agreed to this operation had he known about the consequences. CS did not significantly impair quality of life in the other patients. An inquiry of the patients’ satisfaction with their operations at the end of year 1 showed that 22 patients in group A (88%) and 23 in group B (92%) found it excellent, while 2 in group A (8%) and 2 in group B (8%) found it satisfactory. Only 1 patient in group A (4%) found it unsatisfactory. There was no statistically significant difference between the groups in terms of satisfaction with the operation (*P* = .203).

## 4 Discussion

PH can present at any age, although it tends to affect adolescents and young adults predominantly.^[10]^ Most sweat glands are of the eccrine type, becoming active with puberty, which may explain why hyperhidrosis is rarely seen at an early age and generally becomes evident in the 2nd and 3rd decades of life.^[9]^ Considering the mean ages of the PH patients in this study, they were mostly young and this was compatible with the data given in the literature.^[4,6]^ There were more male patients than females in our study, despite its equal prevalence in both genders. One hypothesis for this is that PH is not seen as a disease among the female population living in the East Black Sea Region. Because of their lower socio-cultural level, they are often unaware of surgical treatment for PH.

The efficacy of ETS in PH was tested with the presence of dryness and increased heat in the hands immediately after the sympathectomy. This study found that all of the patients in both of the groups had dryness and increased heat in their hands after the procedure, with the cessation of their sweating. All of the subjects were discharged with a 100% patient satisfaction. Although there are varying data in the literature for the rate of recurrence after ETS for PH, it is known to range between 1% and 27% and occurs usually within 1 year of surgery.^[2,4]^ In an unsuccessful ETS, a different anatomic structure is often cauterized instead of the sympathetic chain, or an incomplete interruption of the sympathetic chain occurs, and these situations are evidenced by the continuation of the preoperative symptoms during the postoperative period. However, the real recurrences that occur after symptom improvements during the postoperative period are often associated with insufficient administration of the sympathectomy due to the anatomic variability of the sympathetic chain among patients, or the nerve regeneration that may occur in the longer term. One hypothesis for the 100% patient satisfaction and absence of any recurrences in the early period of this study is that after having located the sympathetic chain in both groups the sympathetic chain was cauterized, completely interrupted its continuity, the communicating rami, and any accessory nerve fibers (the nerve of Kuntz). This was achieved by continuing the cauterization for a few centimeters along the rib toward the lateral side.

After an ETS, excessive dryness is seen in the hands of some patients because of complete cessation of sweating. This condition usually does not irritate the patients who suffered from serious personal inconveniences and social problems for years due to PH. Yet, it may necessitate daily use of moisturizing hand creams in some patients with excessively dry hands. Bachmann et al^[12]^ have reported in their study that excessively dry hands in the postoperative period are seen more in patients with axillary hyperhidrosis than those with PH. Wilson et al^[13]^ found in their study that 51% of their subjects had excessively dry hands after an ETS, and this was 6 times more prevalent in females. In this study, excessively dry hands occurred in 3 subjects (6%, 2 females and 1 male), and this dryness did not cause any serious irritation and diminished in time. This condition may be associated with the excessive interruption of impulse transmission from the sympathetic chain to eccrine sweat glands and the loss of functionality in eccrine sweat glands in this region.

CS is an important sign of an unsuccessful surgery as well as a major cause of postoperative dissatisfaction after an ETS for PH.^[10]^ The physiopathology of CS is not fully known, but it is thought that excessive dryness of hands and facial denervation occur in ETSs that are carried out above the T2 ganglion level and involve removal of long segments through broad resections. This in turn is thought to trigger the occurrence of CS. Actually, CS is a combination of a temperature-regulating compensatory mechanism and a reflex response in the body.^[6,7]^ In this study, CS occurred at a rate of 12% in group A and a rate of 8% in group B. Severe CS caused patient dissatisfaction and regret in only one subject in group A. CS was mild in the other subjects and was not at a level to considerably impair their quality of life. When these results are compared to the CS occurrence rates and CS severity after an ETS in the literature, they have lower rates and seem to be satisfactory. In this study, the sympathetic chain and accessory nerve fibers were transected over the 2nd ribs (T2) of group A and over the 3rd ribs (T3) of group B using electrocauterization. Only the sympathetic chain was interrupted with a limitation on the range of dissection, causing no damage to ganglia. The axons from the spinal cord neurons innervating the ganglia were not harmed, by protecting the tissues at the medial sympathetic chain. This approach decreases the synaptic reorganization at the sympathetic chain level.^[1]^ Although the rate of CS occurrence was more in T2 level ETS than in T3 level ETS in this study, the difference was not statistically significant. This indicates that rather than the level of ETS, the procedures carried out to protect the ganglia caused less frequent and severe CS.

CS is a condition in which excessive sweating occurs in the back, waist, groins, and legs of a patient where there was no sweating before the operation. In our study, sweating occurred in the back and abdominal areas of the patients who developed CS and this was compatible with the data in the literature.^[6,11]^

CS developed in 5 subjects in our study, and of these, only one 28-year-old male patient in group A had severely irritating CS in his abdominal region, which caused patient dissatisfaction. This happened in the late postoperative period (month 7), became increasingly severe and then leveled off. It is reported in the literature that the severity of CS may diminish and eventually disappear over time, but this patient has not experienced such a situation to date.^[2,3]^ In general, the severity of CS is inversely correlated with the degree of patient satisfaction.^[10]^ CS was so severe in this patient that he had to change his underwear several times daily and for this reason the patient stated that he regretted having undergone this operation. There is no adequate and appropriate treatment for CS, which is a factor that directly affects the results of an operation and patient satisfaction. Therefore, it is more important at this point to prevent the occurrence of CS rather than treating it.

The 100% overall patient satisfaction obtained in this study in the early period declined to 96% in group A by the end of year 1, but remained at 100% in group B. This rate compares well to the patient satisfaction rates obtained in other studies in the literature.^[11–13]^ The major markers affecting patient satisfaction after an ETS in PH are recurrence and CS.^[2]^ No recurrence was seen in any of the subjects in our study, and quality of life was adversely affected in only 1 of the 5 subjects who developed CS. Resolution of PH symptoms was observed with no recurrence, satisfying the patients and increasing their quality of life score even with CS present. The limitation on the dissection and avoidance of any damage to ganglia produced a satisfactory result by ending the PH sweating and preventing any complications or side effects. This resulted in short and long-term success.

The present study has clear limitations. It is a single-center study and the small number of examined subjects stands at the forefront of these constraints. In addition, the interviews of the patients to reveal their ratings for the success of, and satisfaction with, the procedure were based on subjective components and the follow-up time was short. The results obtained from this study can gain more meaning when further multicenter studies using objective questioning criteria and well-defined validated scales are conducted with a greater number of patients and longer follow-ups.

In conclusion, when an ETS applied at T2 or T3 levels in PH involves only the interruption of the sympathetic chain, with a limitation on the range of dissection and avoidance of any damage to ganglia, sweating is stopped completely, no recurrence is encountered, and CS develops only at low rates and severities. This surgical approach shows tremendous potential in preventing the irritating and feared side effects and complications of ETS, such as recurrence and CS.
